# The COVID-19 pandemic as an disruptive event in school health promotion. Survey results from Germany: Kevin Dadaczynski

**DOI:** 10.1093/eurpub/ckac129.656

**Published:** 2022-10-25

**Authors:** K Dadaczynski, M Messer, O Okan

**Affiliations:** 1 Department of Health Science, Fulda University of Applied Sciences, Fulda, Germany; 2 Center for Applied Sciences, Leuphana University of Lueneburg, Lueneburg, Germany; 3 Department of Nursing Science, Trier University, Trier, Germany; 4 Department of Sport and Health Sciences, Technical University Munich, Munich, Germany

## Abstract

**Background:**

This study examines the extent to which schools implement activities on health promotion and prevention during the COVID-19 pandemic. Moreover, potential differences with regard to demographic variables, school type, state, and participation in state public health and health promotion initiatives are determined.

**Methods:**

As part of the international COVID-Health Literacy Research Network, an online-based cross-sectional study was conducted from March to April 2021 with 2,186 school principals from three German federal states. The implementation status of COVID-19 related school health promotion was assessed using a self-developed instrument. After examining the factorial structure of the instrument, univariate and bivariate data analyses were performed.

**Results:**

Three dimensions of implementing school health promotion can be identified (1. COVID-19- related support for pupils, 2. Health promoting design of teaching, learning and working conditions, 3. Principles of Health Promoting School). A low level of implementation can be observed for aspects of teaching, learning and working conditions (31%) as well as for participation (52%) and cooperation with community stakeholders (42%). Significant differences can be determined with female, older and primary school principals reporting a higher implementation status while for federal state mixed results are found. Stratified by participation in state health promotion initiatives, only schools with a certificate in health promotion show a higher level of implementation.

**Conclusions:**

The results indicate that the COVID-19 pandemic is a disruptive event for schools, impeding the implementation of holistic activities on health promotion and prevention. In addition to systematic support for school principals in the area of health promotion and prevention, it should be ensured that existing initiatives are provided with sufficient resources, especially in times of crisis.

